# The Demographic-Wealth model for cliodynamics

**DOI:** 10.1371/journal.pone.0298318

**Published:** 2024-04-02

**Authors:** Lukas Wittmann, Christian Kuehn

**Affiliations:** Department of Mathematics, School of Computation Information and Technology, Technical University of Munich, Garching, Germany; University of Aberdeen, UNITED KINGDOM

## Abstract

Cliodynamics is a still a relatively new research area with the purpose of investigating and modelling historical processes. One of its first important mathematical models was proposed by Turchin and called “Demographic-Fiscal Model” (DFM). This DFM was one of the first and is one of a few models that link population with state dynamics. In this work, we propose a possible alternative to the classical Turchin DFM, which contributes to further model development and comparison essential for the field of cliodynamics. Our “Demographic-Wealth Model” (DWM) aims to also model link between population and state dynamics but makes different modelling assumptions, particularly about the type of possible taxation. As an important contribution, we employ tools from nonlinear dynamics, e.g., existence theory for periodic orbits as well as analytical and numerical bifurcation analysis, to analyze the DWM. We believe that these tools can also be helpful for many other current and future models in cliodynamics. One particular focus of our analysis is the occurrence of Hopf bifurcations. Therefore, a detailed analysis is developed regarding equilibria and their possible bifurcations. Especially noticeable is the behavior of the so-called coexistence point. While changing different parameters, a variety of Hopf bifurcations occur. In addition, it is indicated, what role Hopf bifurcations may play in the interplay between population and state dynamics. There are critical values of different parameters that yield periodic behavior and limit cycles when exceeded, similar to the “paradox of enrichment” known in ecology. This means that the DWM provides one possible avenue setup to explain in a simple format the existence of secular cycles, which have been observed in historical data. In summary, our model aims to balance simplicity, linking to the underlying processes and the goal to represent secular cycles.

## 1 Introduction and modelling background

Although Cliodynamics [1] is a rather new research area, there are already several interesting theories regarding the interplay of state and population dynamics. To derive a model, we first briefly review and examine basic theories of state and population interplay to illustrate the multitude of possible approaches that have been proposed. In fact, this diversity of approaches justifies to also try out different mathematical models, which is the route taken in this work.

We only review theories briefly to indicate their diversity and complementary aspects. Turchin states, that empires typically can be in three different stages during their existence [2, p. 133]:

(T1) Polity formation and ethnogenesis, accompanied by initial expansion within an ethnically similar substrate.
(T2) Expansion to peak size, in the process acquiring a multiethnic character.
(T3) Stagnation, decline, and collapse (perhaps followed by a revival, and another imperial cycle).

Although (T1)-(T3) are theoretical assumptions that could be used as the basic for a mathematical model for the rise-and-fall of states, there are certainly many nuanced factors that one may want to take into account for this process. According Khaldun [3] there are two major causes for state collapse, ideological and economic. The according theory, [3, p. 355], can be roughly summarised by the following statements:

(K1) Recently established states are moderate in expenditure and just in administration, resulting in light taxation.
(K2) Under the circumstances (K1), population numbers are rising.
(K3) Prosperity leads to increased spending to maintain wealth.
(K4) Higher wealth demands higher expenditures for state security and bureaucracy.
(K5) Population numbers eventually reach a growth limit.
(K6) Maintaining prosperity without population growth leads to higher taxation and exploitation of the residents.
(K7) Higher taxation may ruin the economy and eventually leads to famines, rebellion or political unrest.

Observe that one view of the progression of events (K1)-(K7) is already quite a fine-grained view of secular cycles. Yet, the more economically focused explanation by Khaldun is just one possibility to explain the detailed cycle of rise-and-fall for states and populations. Another complementary theory is described by Goldstone [4, p. 24]. It centers around the idea that population growth causes social crisis indirectly, affecting social institutions, which in turn affect social stability. This means that if the population grows in excess of the productivity gains of the land, there are multiple effects on social institutions:

(G1) Excessive population growth leads to inflation which may prevent or reduce tax revenues due to economic uncertainty.
(G2) Increased population leads to expansion of armies and rising real costs.
(G3) Increased population leads to rural misery, urban migration and famines.
(G4) Increased population leads to expansion of youth cohorts, often impacted by lack of employment opportunities.
(G5) Rising costs and low revenues lead to high taxation.

The final outcome of this theory is similar to the one of Khaldun, state bankruptcy, loss of military control and rebellion. Yet another variant for this process can be based on considerations by Olson [5], which is a social-economic hybrid explanation. The theory states that rapid economic growth means rapid economic change which entails social dislocation. Both gainers and losers from economic growth can be destabilizing. More precisely, the steps considered by Olson are:

(O1) Economic growth increases the number of “nouveaux riches”.
(O2) Economic growth also creates a large number of “nouveaux pauvres”.
(O3) The “nouveaux riches” can use the gained power to change social and political order in their interest.
(O4) The “noveaux pauvres” will be more resentful of their poverty than those who have known nothing else.
(O5) Individuals gain economic power incompatible with their positions in the social and political order before.
(O6) That new power can be used in their own interest to change the political or social order.

Based on (O1)-(O6), and observing that the economic, social and political systems are clearly interdependent parts, a quick change in one part may lead to instability in other parts of the society. This can lead to a change in social and political order that is suited to the new distribution of economic power. But as the growth is very rapid, the path to this new equilibrium may be very unstable [5, p. 533]. Huntington [6], concurred with Olson’s theory and asserts that fast economic growth and the rapid political change that often follows heightened expectations are destabilizing factors, especially if the inclusion of new political participants into the new political system is too slow. Implicitly, both Olson and Huntington are arguing that fast growth in market dynamics leads to dis-integrative behavior when the political or administrative power grows too slow or is low. This leads to destabilisation of the existing system.

The last theory we want to briefly mention here is described by Olson [7] and Collins [8]. It deals with the effects of increased population size. Despite the fact that increased population can improve geopolitical condition because a larger amount of soldiers and labor is available, it also rises the per capita military cost. Therefore, a bigger population is only an advantage if the “competitor’s” situation regarding population and technology remains the same. Also, despite the possibility that increased population can be transferred into more power of the state, the state also has to incorporate and protect the expanded population. Larger population also means that there is a need of more living space which makes the states territory more difficult and more expensive to protect and to rule. This can again lead to destabilisation of the state with a possible breakdown.

In summary, the various theories in cliodynamics that could be used as a basis to explain the rise-and-fall of states are highly complex and often describe complementary potential factors for state-rise and state-collapse. Once one goes beyond basic very macroscopic (in space and time) principles such as (T1)-(T3), there are various options available that may be plausible and complementary. The examples we have given here regarding (K1)-(K7), (G1)-(G5) and (O1)-(O6) are certainly not exhaustive. Furthermore, giving a pure verification from a data-analytic viewpoint of certain theories can be complicated as the systems are highly complex and data has to be gathered on extremely long time scales to obtain reliable statements about human behaviour during rise-and-fall of states. Such a situation is not uncommon in many other sciences, e.g., climate science and ecology, face a similar dilemma. Hence, using mathematical modelling and simulation tools can greatly help to explore the space of possibilities and explanations. This is the viewpoint we take in this paper.

However, this triggers the question: How to mathematically model the theories we recalled above? Suppose we agree on a macroscopic model only including the main observables, which already discards quite substantial issues such as complex network coupling between agents. Then we should start to list the main time-dependent observables and ask, how they depend on the economic, social, and other factors that we outlined in the theories above. Table 1 shows some possible macroscopic variables that one might consider and how they are influenced by certain events. The table clearly shows that it is already extremely difficult to decide, (a) which macro-variables to select and (b) how to even model their qualitative changes for certain events. For example, suppose we have a change in political power. What would this entail? This is evidently unclear and depends very much on the historical and political circumstances. Therefore, many existing mathematical models in cliodynamics have taken the approach to limit the dynamics to a very small set of macro-observables such as population size and state wealth. Although this very likely overly simplistic, it at least gives an idea of which effects are *possible*. Therefore, we have to keep in mind that validating effects against limited historical data will in most cases be *insufficient to directly match* the model with a particular situation. Nevertheless, starting with simple models can clearly help us to understand certain patterns better and slowly improve our understanding, what the crucial ingredients of a more precise theory should be.

**Table 1:** This table lists a possible, yet certainly very much up to debate, attempt to identify some time-dependent macro state variables (leftmost column) and how they might depend upon certain observed processes/events such as population growth, hitting a situation close to carrying capacity, high state costs, rebellions/wars, changes in political power, etc. Signs indicate, whether the event/process might positively (+) or negatively (-) change the dynamics of the macro-variables. We clearly observe that there could be many many more possible variables and that already the positive/negative influence modelling is difficult, which does not even bring up the matter of possible functional form relationships to express the model precisely.

Possible Modelling Ingredients/Events						
Macro-Variable	Population Growth	Carrying Capacity	High State Costs	Rebellions & Wars	Power Change	⋯
Population Size	+	-	+	-	+/-	
State Wealth	+	-	-	-	+/-	
Taxation Rate	+	-	+	+	+/-	
Nouveaux Riches	+	+/-	+/-	-	+/-	
Nouveaux Pauvres	+	+	-	-	+/-	
Army Size	+	-	-	+	+/-	
Social Balance	-	-	+	-	+/-	
⋮						⋱

In this work, we are going to start with a model, the “Demographic-Fiscal Model” (DFM), introduced by Turchin and then use the previous modelling from this introduction to develop a variant of this model, which we call the “Demographic-Wealth Model” (DWM). We emphasize that our approach to propose another model is simply motivated by the fact that the social, political and economic theories behind the initial models proposed in cliodynamics are extremely diverse. We want to reflect this diversity also on the side of the mathematical models and this motivated us to introduce the DWM. In the important technical part of this work, we are going to use mathematical tools from nonlinear dynamics to analyze the DWM in more detail. We believe that these tools can also be useful for other models in cliodynamics to explore modelling options and parameter spaces.

## 2 The classical “Demographic-Fiscal model”

### 2.1 Derivation of the DFM

The “Demographic-Fiscal model” (DFM) was derived by Peter Turchin in 2003 [2]. Turchin pays attention to a particular possible correlation, a feedback effect of political instability on population dynamics. He concludes that political instability can negatively affect both demographic rates and the productive capacity of the society, see also [9]. The goal of the DFM is to derive a model in which population dynamics is an endogenous process, not only consisting of the link between population growth and state breakdown. Turchin considers the economic factor of state decline and mainly uses two theories, the ones by Khaldun and Goldstone, presented briefly in Section 1. The mathematical model consists of two variables, the population density *N*(*t*) and the accumulated state resources *S*(*t*), measured in grain,

$$\begin{array}{c} \begin{array}{cc} \dot{N} & =rN(1-\frac{N}{k(S)}),\\ \dot{S} & =\rho_{0}N(1-\frac{N}{k(S)})-\beta N, \end{array} \end{array}$$ (1)

where *r*, *ρ*_0_, *β* > 0 are parameters. The functional form of *k*(*S*) implies that a strong state has a positive effect on population dynamics. More precisely, the “carrying capacity” is an increasing function of *S*. As *k* cannot increase without bound, because at some point all available land and space is used and maximum productivity is reached, there has to be a bound *k*_*max*_. This yields

$$\begin{array}{c} k\left(S\right)=k_{0}+c\frac{S}{s_{0}+S}. \end{array}$$

Here *k*_0_ is the carrying capacity of a stateless population, *c* = *k*_*max*_ − *k*_0_ is the maximum possible gain in increasing *k* and *s*_0_ indicates how the improvement in *k* depends on *S*.

### 2.2 Analysis of the DFM

The analysis provided below mainly deals with the dynamics and the stationary states of the DFM as well as the discussion of important consequences of the derivation and the analysis provided by Turchin. An important condition for the analysis of model (1) is that the state is not allowed to get into dept, leading to the condition *S* ≥ 0. With all parameters being positive we first take a look at possible stationary states of the system. Therefore, we need

$$\begin{array}{cc} 0 & \overset{!}{=}rN(1-\frac{N}{k(S)}),\\ 0 & \overset{!}{=}\rho_{0}N(1-\frac{N}{k(S)}-\frac{\beta }{\rho_{0}}). \end{array}$$

Aside from the trivial state *N* = 0 where no population is present, it is clear that system (1) in its natural form cannot have any additional equilibrium points. But with the criterion *S* ≥ 0, which we can enforce by setting the time derivative of *S* equal to zero once *S* reaches zero, an additional stationary state can be found, namely (N*, S*) = (*k*_0_, 0), which is locally stable. The equilibrium only exists because it is assumed that as soon as *S* would become negative after some time t* the state collapses, so *S* is set to 0. Setting *S* = 0 results in the system

$$\begin{array}{cc} \dot{N} & =rN(1-\frac{N}{k_{0}}),\\ S & =0, \end{array}$$

after the time t*. Looking at the first equation, $\dot{N}=0$ yields the stationary state (N*, S*) which is obviously stable. The outcome of Turchin’s model in general is that once a state has risen it is determined to vanish after a certain time span. The only possibility for another cycle to form is a perturbation of the system, so either *N* has to be decreased or *S* has to be increased.

### 2.3 Discussion of the DFM

In the derivation of the dynamics for *N* it is assumed that the per capita rate of population increase is a linear function of the per capita rate of surplus production, yielding the logistic model for population growth. For the dynamics of the state, the same approach is used with other parameters, which is a reasonable explanation.

**Fig 1:**
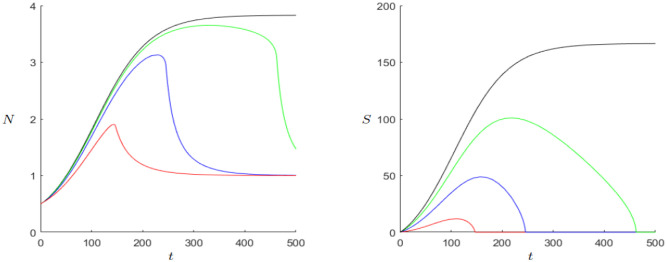
Dynamics of model (1). Initial Values: *N*_0_ = 0.5, *S*_0_ = 0. Parameters: *k*_0_ = 1, *r* = 0.02, *ρ*_0_ = 1, *c* = 3, *s*_0_ = 10. Values for *β*: red ≡ 0.4, blue ≡ 0.25, green ≡ 0.1, black ≡ 0.

**Fig 2:**
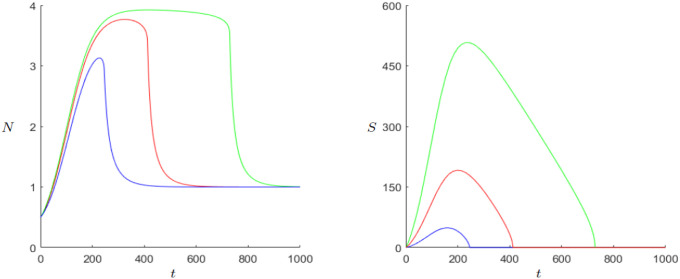
Dynamics of model (1). Initial Values: *N*_0_ = 0.5, *S*_0_ = 0. Parameters: *k*_0_ = 1, *r* = 0.02, *β* = 0.25, *c* = 3, *s*_0_ = 10. Values for *ρ*_0_: blue ≡ 1, red ≡ 2, green ≡ 4.

We take a look at the results of the DFM in Figs 1 and 2. Take *β* = 0.25 (blue line) as the original expenditure rate. For values greater than 0.25 (red line), which means more expenditures for the state, the result would be negative for both, state and population. Higher expenditures lead to a shorter and less wealthy lifetime of the state while the population approaches its equilibrium *k*_0_ sooner. As an alternative, one might want to model the case that a higher expenditure rate should be beneficial for the population. For values smaller than 0.25 (green and black line) the outcome of the system is more beneficial for both, the state and the population. In case of the state this would make sense as lower expenditures would yield a higher surplus, but one might want another possible outcome for the population. The extreme case *β* = 0 would lead to the situation where the population would approach its maximum *k*_*max*_ and the state would never collapse. Hence, the model takes a quite stabilizing view on low state expenditure for the population. Yet, one might also want to consider alternative models, where the feedback situation between expenditure and population size is different.

Another important effect in the DFM is the modelling of the response from *S* to *N*. The feedback effect from *S* to *N* through *k*(*S*) is a positive one, increasing the carrying capacity for the population. This is definitely a valid approach but alternatively one could aim to model the negative effect of excessive taxation, which might be bad for the population, especially if the taxes are far too high or if the taxes are counter-productive due to their structure. In other words increasing the tax rate may only be beneficial for the population up to a specific value. For the system (1) for different values of *ρ*_0_ it can be seen that an increase of the tax rate is beneficial for both, the state and the population. Indeed, the population will at some point break down again to its equilibrium and the state will also collapse at some point but in the time between the population reaches a higher level and lasts longer, as well as the state. This illustrates the different modelling choices and interpretations that are put into the DFM model as well as the wide variety of possible approaches to modelling taxation [10].

An extensive analysis of the DFM is also done by Maini [11]. To summarize, the main outcome of the DFM is a stateless society, where the population stays at its starting value *k*_0_. In other words, the long-time outcome of the system is the starting point of the system, which is still reasonable if one is interested in the transient dynamics and uses re-initalization of the system. Yet, one could also aim for alternative models, where periodicity is immediately built in to explain the recurring theme of rise-and-fall of states. In particular, one may ask, whether there is a simple alternative model with slightly different assumptions that leads to periodicity.

## 3 The “Demographic-Wealth model”

### 3.1 Model derivation

The main difference in the interpretation of the model compared to the DFM is the state’s role in it. In the DFM the state’s surplus is determined through revenues and expenditures, measured in taxes, respectively more money that has to be spent in order to maintain the state’s infrastructure with growing population numbers. In our “Demographic-Wealth model” (DWM) the state’s surplus/wealth is measured in wealth gain and wealth loss and the gains are mainly determined by two aspects:

- Taxes that are collected from the population.
- Wealth that is generated with existing surplus, for example land gain through warfare, trade or strategic investments.

In addition, the wealth loss is mainly on the state’s wealth level. The more wealth the state has (more money or more land and therefore gaining more attention), the more expenditures it has to make in order to secure this wealth (from attacks, land loss and maintaining infrastructure), similar to the theory of Olson. Starting with the dynamics for *N*, as in the DFM, a logistic growth for the population is assumed, with *r* being the intrinsic rate of population growth, so in absence of the state, the population will grow until its “carrying capacity” *k*. The carrying capacity *k* is a functional response to the state’s wealth,

$$\begin{array}{c} k\left(S\right)=k_{0}+cS, \end{array}$$ (2)

meaning that more wealth and therefore more land and financial possibilities lead to more space and resources to live. The parameter *k*_0_ is the carrying capacity in absence of the state and *c* determines the dependence of the carrying capacity on the change in state’s wealth. The difference to the functional *k*(*S*) in the DFM comes from the interpretation of *S* as the wealth of the state (land gain). A new part for the dynamics of *N* is the a negative feedback effect from *S* to *N*, inspired by Olson. A state that is growing in wealth has at some point negative influence on population numbers, for example, one may consider the scenarios:

- Growing wealth leads to growing expenditures which lead to exploitation of the population.
- Growing wealth leads to more warfare and therefore to a higher death rate or emigration.

In addition, with growing population and only a limited amount of food and living space available and taxes that have to be paid, a growing fraction of the population cannot afford living in the state. So, there will be a growing fraction of the population that leaves the state or dies. On the other hand, the remaining people have more resources and space, so it is assumed that the decreasing rate approaches one. Together with the negative feedback effect from *S* to *N* this behavior is described by the term −*αX*(*N*, *S*). In this model the functional $X\left(N,S\right)=\frac{SN}{d+N}$ is chosen, because of the situation described above, where *d* > 0 controls the strength of the negative feedback from *S* to *N* in the usual way of a Holling type-II response. Overall, the dynamics of *N* has the form

$$\begin{array}{c} \dot{N}=rN(1-\frac{N}{k(S)})-\alpha S\frac{N}{d+N}. \end{array}$$ (3)

The dynamics of *S* are determined by wealth gain and wealth loss of two parties, the population and the state itself. The state collects taxes and can reinvest a portion of the surplus gained through the population for some extra wealth, for example through loans, warfare or land gain. This results in the term *gSN*, with *g* = *τρ*, *τ* being the tax rate and *ρ* the fraction of the surplus that is gained through investing/expanding. But there are also expenditures that the state has to make. The larger the country and the more wealth the state has, the more money it has to spend for protection or maintaining the wealth. For example, if the state gains wealth through capturing new land, it has to pay additional attention to protect the new land by paying more soldiers and civil servants. In addition, it needs to provide a suitable living space, so it has to reinvest a growing amount of money into the infrastructure of the country. In summary, we consider that the dynamics of *S* has the form

$$\begin{array}{c} \dot{S}=gSN-\beta S, \end{array}$$ (4)

with *β* being the fraction of the wealth that has to be spent. Together with the Eqs (2) and (3) the “Demographic-Wealth model” (DWM) is given by

$$\begin{array}{c} \begin{array}{cc} \dot{N} & =rN(1-\frac{N}{k_{0}+cS})-\alpha S\frac{N}{d+N},\\ \dot{S} & =gSN-\beta S. \end{array} \end{array}$$ (5)

Note that different choices for the interaction functions between population and state are definitely possible, e.g., the nonlinearities may also carry different powers for *S* and *N*. Here we have effectively applied the principle to start with the simplest non-trivial case of a direct production interaction leading to the terms *SN*.

### 3.2 Model analysis

For a theoretical background regarding stability and bifurcations we refer to the introductory textbook [12] and the more advanced monographs [13, 14]. Looking for possible stationary states the following equations have to be satisfied

$$\begin{array}{c} \begin{array}{cc} 0 & \overset{!}{=}[r(1-\frac{N}{k_{0}+cS})-\alpha \frac{S}{d+N}]N\\ 0 & \overset{!}{=}(gN-\beta )S. \end{array} \end{array}$$ (6)

One can see that (0, 0) and (*k*_0_, 0) are stationary states. For a possible coexistence point (N*, S*) it follows that $N^{*}=\frac{\beta }{g}$ and S* has to satisfy

$$\begin{array}{cc} \alpha \frac{S^{*}}{d+N^{*}} & =r(1-\frac{N^{*}}{k_{0}+cS^{*}}). \end{array}$$

With the calculations for the Jacobian and its determinant and trace the stability behavior of the stationary states can be determined. For (0, 0) one has det(J(0,0))=-rβ which yields a saddle point and therefore (0, 0) is always unstable. For the stationary state (*k*_0_, 0) the following can be calculated:

$$\begin{array}{cc} \text{det}(J\left(k_{0},0\right)) & =r(\beta -gk_{0}),\\ \text{tr}(J\left(k_{0},0\right)) & =-r-k_{0}g-b. \end{array}$$

It follows that $\frac{\beta }{g}>k_{0}$ yields a stable equilibrium (*k*_0_, 0), more precisely a sink. If $\frac{\beta }{g}<k_{0}$ the equilibrium is a source, therefore unstable. For the analysis of the coexistence point the following functions are introduced.

$$\begin{array}{cc} g(N,S) & =r(1-\frac{N}{k_{0}+cS}),\\ p(N) & =\alpha \frac{N}{d+N},\\ q(N) & =gN. \end{array}$$

One can then show the following result:

**Theorem 4.1**. *Take g*(*N*, *S*), *p*(*N*) *and q*(*N*) *as described above and let* (N*, S*) *be the coexistence point. Let*

$$\begin{array}{c} p\left(N^{*}\right)>N^{*}\frac{\partial }{\partial S}g\left(N^{*},S^{*}\right). \end{array}$$

*Introduce*

$$\begin{array}{c} \mu \left(N^{*},S^{*}\right)=p\left(N\right)\frac{\partial }{\partial N}(\frac{Ng(N,S^{*})}{p(N)}){|}_{N=N^{*}}. \end{array}$$

*If μ*(N*, S*) < 0, *then* (N*, S*) *is stable. In addition, all parameter combinations that yield μ*(N*, S*) = 0 *correspond to a Hopf bifurcation of* (N*, S*).

For a proof, we refer to Appendix A. In fact, in Appendix A, we also give a more general description of the phase plane analysis of the DWM within the proof. Unfortunately Theorem 4.1 is rather implicit and is difficult to apply in practice as one would prefer a result with more explicit parameter dependence. For a special case, the result becomes more transparent. If we assume that *c* = 0 the state’s wealth no longer influences the carrying capacity. In reality this could for instance happen if there is simply no additional room left (an island for example). Now, the stability behavior of the equilibria (0, 0) and (*k*_0_, 0) do not depend on the parameter *c* and we get a more explicit result:

**Theorem 4.2**. *Let c* = 0, $k_{0}>2\frac{\beta }{g}$, *d* < *k*_0_. *Then Hopf bifurcations occur in the DWM whenever*

$$\begin{array}{c} k_{0}=2\frac{\beta }{g}+d,\;\;\;\;d=k_{0}-2\frac{\beta }{g},\;\;\;\;\beta =\frac{1}{2}\left(k_{0}-d\right)g,\;\;\;\;g=\frac{2\beta }{k_{0}-d}, \end{array}$$ (7)

*i.e., periodic solutions are generated at the Hopf bifurcation point*.

The proof and a more detailed phase plane analysis can be found in Appendix B. We note that the detailed mathematical analysis of the dynamics will likely be very difficult if one considers varying through all possible parameter configurations. Yet, the main conclusion from Theorems 4.1-4.2 is that the DWM can naturally yield periodic behaviour without any resets. Now, we have to check whether our dynamical analysis is consistent with the intended interpretation/modelling from the theories underlying cliodynamics. Yet, we already emphasize that any low-dimensional model of such a high-dimensional complex system such as state/population models must necessarily simplify and can only depict certain aspects of the overall dynamics.

### 3.3 Interpretation of different behaviors

Starting with the equilibrium (*k*_0_, 0) the criterion for stability is $\frac{\beta }{g}>k_{0}$. For the simple case *k*_0_ = 1, this means that if the expenditures *β* exceed the “growth-rate” *g* of the state then the state cannot survive and the stateless population approaches its carrying capacity. Another interpretation could be that the carrying capacity *k*_0_ is too small in comparison to a given expenditure/growth-rate ratio for a state to survive.

**Fig 3:**
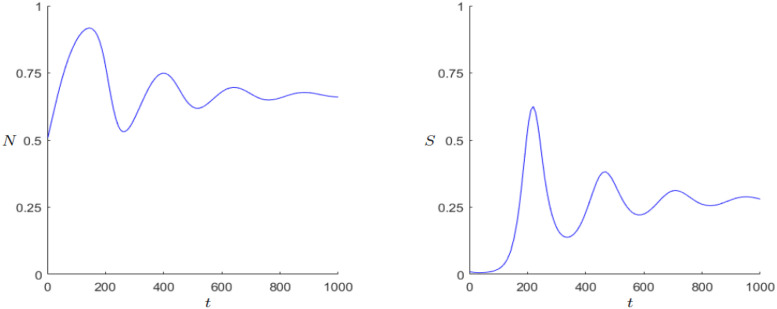
Dynamics of the “Demographic-Wealth model” with stable equilibrium (N*, S*). Parameters: *r* = 0.02, *g* = 0.15, *a* = 0.05, *b* = 0.1, *c* = 0.5, *k*_0_ = 1, *d* = 1. Initial values: *N*_0_ = 0.5, *S*_0_ = 0.01.

We have also used numerical continuation of the equilibrium to track its stability using the software MatCont, see [15] for details. The numerical continuation runs, reported in more detail below, for the equilibrium (*k*_0_, 0) is consistent with the analysis provided above, meaning that for the parameters *g*, *β*, and *k*_0_ it shows a loss of stability whenever $\frac{\beta }{g}<k_{0}$. Following the analysis above, the equilibrium (N*, S*) becomes stable as the equilibrium (*k*_0_, 0) becomes unstable. The typical behavior is shown in Fig 3. Both the population numbers and the state’s wealth exhibit damped oscillations until the coexistence point is reached.

**Fig 4:**
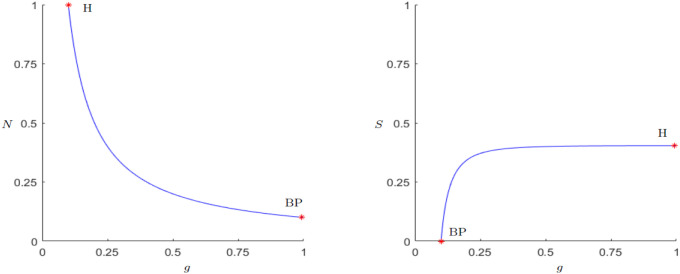
Equilibrium point continuation for the parameter *g*. Existence of a Branch point at *g* = 0.1 where the equilibrium (N*, S*) becomes stable. Supercritical Hopf bifurcation at *g* = 0.992, 1st Lyapunov coefficient <0.

**Fig 5:**
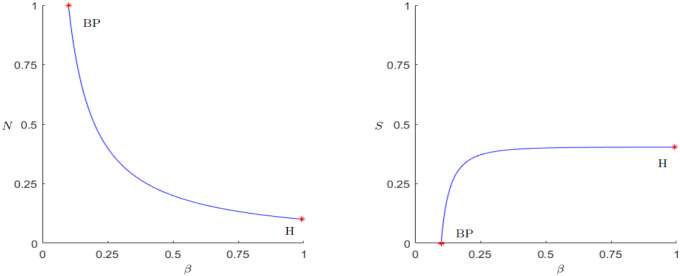
Equilibrium point continuation for the parameter *β*. Existence of a Branch point at *β* = 0.15 where the equilibrium (N*, S*) looses its stability. Supercritical Hopf bifurcation at *β* = 0.015, 1st Lyapunov coefficient <0.

**Fig 6:**
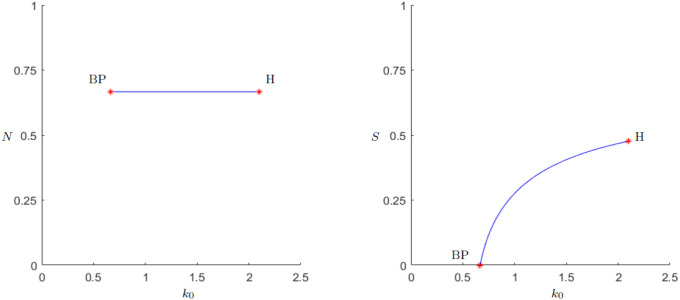
Equilibrium point continuation for the parameter *k*_0_. Branch point at $k_{0}=\frac{2}{3}$ where the equilibrium (N*, S*) becomes stable. Supercritical Hopf bifurcation at *k*_0_ = 2.095, 1st Lyapunov coefficient < 0.

As described in the analysis of the coexistence point, there are two possible ways for a Hopf bifurcation to occur, either making N* small enough, or changing S* in a way that *μ*(N*, S*) is equal to zero. In the case of N* there is the possibility to chose *g* big enough, *β* small enough or changing *k*_0_. Checking the numerical continuations shown in Figs 4–6, indeed a Hopf bifurcation occurs for both parameters, at *g* = 0.992 and *β* = 0.015, both rounded to 3 decimal digits. By calculating *μ*(N*, S*) for these sets of parameter values one gets approximately zero in both cases.

For the remaining parameters of the model *r*, *α* and *c*, the continuation also shows Hopf bifurcations for certain parameter values. This corresponds to an increase in S* yielding *μ*(N*, S*) = 0.

### 3.4 Interpretation of the DWM

We want to look at the parameters of the “Demographic-Wealth model” and whether their change induces meaningful dynamics based upon their interpretation, starting with the three most important parameters, *g*, *β* and *k*_0_. As a starting point, a stable equilibrium (N*, S*) is assumed. For the parameter *g* one can see in Fig 4 that for values between 0.1 and 0.992 the equilibrium remains stable. A smaller parameter *g* results in higher population numbers and lower state’s wealth, which results from lower taxes. If *g* < 0.1, (N*, S*) and (*k*_0_, 0) swap stability, meaning the state cannot survive. One explanation for this behavior is, that the state collects too little taxes, respectively the growth is too small so it cannot survive. On the other hand a greater parameter *g* results in greater wealth of the state but lower population numbers up to a point where (N*, S*) becomes unstable and a limit cycle occurs, meaning a repeated sequence of state growth and state decline. This could happen because if taxes are too high or growth is too fast, population numbers are plummeting rapidly, and the state cannot compensate the tax loss.

The behavior for the parameter *β* is the other way around, meaning if the state has high expenditures, it benefits the population until the state breaks down, and if the expenditures are low, population numbers are shrinking to the point where the state cannot compensate the loss of population and a limit cycle occurs again at *β* = 0.015.

In case of the parameter *k*_0_, Fig 6 shows that if the carrying capacity is too low ($k_{0}<\frac{2}{3}$) the state has no chance of surviving because the starting capacity is too small. For higher *k*_0_, ($\frac{2}{3}<k_{0}<2.095$), a greater *k*_0_ results in greater wealth for the state whereas the population number remains unaffected. On the other hand, if *k*_0_ is too big (*k*_0_ > 2.095) it leads again to the occurrence of limit cycles. In contrast to the parameters *g* and *β* the occurring limit cycles approach zero for *N* and *S* way closer, so it can be seen as a sequence of state growth and collapse, same holds for the population. Similar limit cycle behavior as for *k*_0_ can only be seen for the parameters *r* and *α*. For the other parameters either the limit cycle is further away from zero or the values for *N* and *S* are not representative anymore.

In summary, we argued that the behavior of the model regarding the three parameters is quite consistent with the theory of Olson described in Section 1, as rapid economic growth (similar to growing *g*) or slow adaption to new situations (similar to small *β*) both can destabilize the state. In addition, the limit cycle behavior for the parameter *k*_0_ coincides with Olson’s theory that a large territory can also lead to state decline due to feedback. Furthermore, we observe that the occurrence of Hopf bifurcations with limit cycles that approach zero very fast is comparable with a similar behavior in biology, the so-called paradox of enrichment.

#### 3.4.1 Paradox of enrichment for the DWM

The paradox of enrichment is a theory from population ecology first described by Rosenzweig in 1971 [16]. In general, enrichment of resource in a predator-prey model [17, p. 111ff] leads to destabilization of the system, leading to collapsing predator and prey population [18, p. 421]. More precisely, Rosenzweig showed that if the carrying capacity of the prey population is increased sufficiently, the coexistence state becomes unstable and the system exhibits limit cycles. Further increase of the carrying capacity leads to growing cycles that approach zero more and more, meaning it can lead to extinction upon small stochastic fluctuation once the limit cycle is close to the zero population level [18, p. 421].

However, this behavior has rarely been observed in real ecosystems [19], and could not be proven in several experiments [20, 21]. Because the “Demographic-Wealth model” is quite similar to a predator-prey model, it should be checked whether such a paradox occurs in the DWM too. Indeed, from the analysis above there appear Hopf bifurcations for the parameters *r*, *α* and *k*_0_ which yield the same behavior as for the predator prey models and therefore for such a paradox.

**Fig 7:**
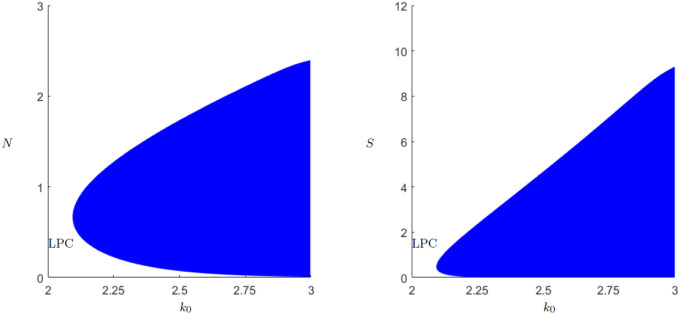
Periodic orbit continuation for the parameter *k*_0_. Continuation of the Hopf bifurcation at *k*_0_ = 2.095, as in Fig 6. The amplitude of the periodic orbit is growing upon increasing the carrying capacity and it eventually comes close to zero population level. In particular, we have the hallmarks of the paradox of enrichment.

**Fig 8:**
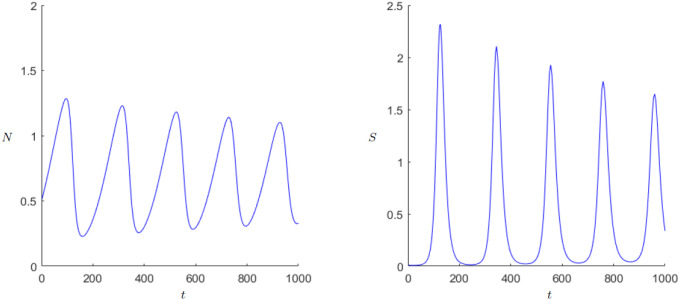
Dynamics of the Demographic-Wealth model with stable equilibrium (N*, S*). Parameters: *r* = 0.02, *g* = 0.15, *a* = 0.05, *b* = 0.1, *c* = 0.5, *k*_0_ = 2, *d* = 1. Initial values: *N*_0_ = 0.5, *S*_0_ = 0.01.

Because in the original predator-prey model it is the carrying capacity that leads to such a paradox, the behavior resulting of *k*_0_ is examined first. Looking at the continuation in Fig 7, one can see that as soon as the Hopf-point is reached the limit-cycles approach zero very fast. To find a possible explanation for the behavior a look at the situation before the Hopf bifurcation is helpful. For *k*_0_ = 1 the behavior is already shown in Fig 3. For *k*_0_ = 2, shortly before the Hopf-bifurcation, we take a look at Fig 8.

Although (N*, S*) is stable in both cases, it takes way longer with growing *k*_0_ until the equilibrium is reached. In addition, the differences between maximum and minimum become greater. Especially for *S*, the minimum is almost approaching zero despite the fact that the equilibrium for *S* increases. One possible explanation for this behavior could be that with greater carrying capacity there is more room to grow fast but the population cannot grow with the same rate as the state’s wealth does. To hold the growth the state has to collect more and more resources per capita until the population cannot endure it anymore and collapses. With that being the case, the state loses its most important support and breaks down. In case of the limit cycle, the carrying capacity becomes too big for the state to establish an enduring society and finds itself in a cycle of growth and collapse. Another explanation for the behavior could be the already mentioned theory of Olson.

For the parameter *r* it is also worthwhile to examine the behavior shortly before the Hopf bifurcation and compare it with the initial parameter combination in Fig 3.

**Fig 9:**
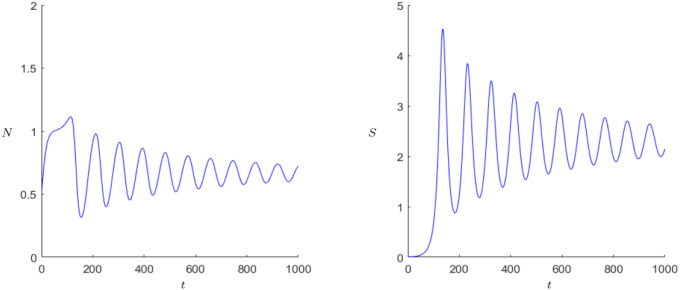
Dynamics of the Demographic-Wealth model with stable equilibrium (N*, S*). Parameters: *r* = 0.1, *g* = 0.15, *a* = 0.05, *b* = 0.1, *c* = 0.5, *k*_0_ = 1, *d* = 1. Initial values: *N*_0_ = 0.5, *S*_0_ = 0.01.

**Fig 10:**
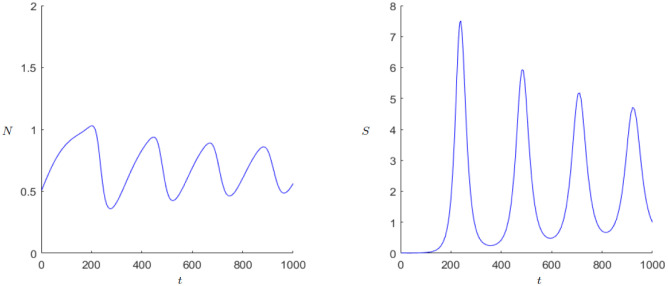
Dynamics of the Demographic-Wealth model with stable equilibrium (N*, S*). Parameters: *r* = 0.02, *g* = 0.15, *a* = 0.01, *b* = 0.1, *c* = 0.5, *k*_0_ = 1, *d* = 1. Initial values: *N*_0_ = 0.5, *S*_0_ = 0.01.

When comparing Figs 9 and 10, we notice that the sum of oscillations increases greatly with growing *r*. This could be a reason for the faster population growth. In addition, the amplitude of the oscillations grow with greater *r*. One possible explanation of the Hopf bifurcation and the series of growth and breakdowns could be that excessive population growth leads to fast state growth but when the population growth starts shrinking again due to lack of space, the state cannot hold up its fast gained wealth and starts decaying.

The last parameter one should look at is *α*, which describes the negative influence from *S* to *N* and of *N* to itself. Unlike the parameter *r* the main behavior that changes with smaller *α* is the amplitude, especially for *S*, as Fig 10 shows. The state can gain more wealth before it declines again. This could be due to the fact that if the negative influence of *S* on *N* shrinks, the state can gain wealth longer before the shrinking population has an effect. If *α* becomes too small, one could say the state uses the little negative influence and starts acting recklessly regarding the population, which again leads to a series of breakdowns and growth.

All of the statements above should be viewed as explorative considerations. Making an experiment in order to clarify if such paradoxes exist in reality is simply not possible on a suitable time scale, let alone on ethical principles. Also looking at old records and comparing it with the considerations is difficult as there are many factors that influence state dynamics, which cannot be ignored. But as there can be limit cycles in the model, it should checked whether these can be found in data, which seems to be indeed the case.

#### 3.4.2 Secular cycles

**Fig 11:**
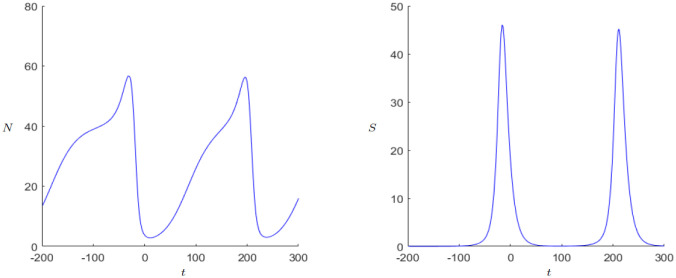
Dynamics of the Demographic-Wealth model with limit cycle and adapted axes. Parameters: *r* = 0.04, *g* = 0.16, *a* = 0.006, *b* = 0.1, *c* = 0.5, *k*_0_ = 1, *d* = 1. Initial values: *N*_0_ = 0.33, *S*_0_ = 0.01.

The pattern of population change is strongly affected by the scale at which it is observed. Population numbers can fluctuate in a span of years for example through bad harvests caused by bad weather. But when considering a longer time scale, decades or centuries, one tends to observe a dominant pattern called Secular Cycles. These cycles are determined by long periods of population growth followed by years of decline. More theory can be found in the work of Turchin and Nefedov [22]. A well-known example for these cycles is Western Europe from the thirteenth to the eighteenth century when there was a growth period in the thirteenth, a decline period in the fourteenth and fifteenth century and again a period of growth in the sixteenth followed by a decline and stagnation in the seventeenth century [1, p. 175]. An application can be found in the work of Alexander, [23]. Another example where comparatively a lot of historical data is available is the Chinese history, since a detailed population history was published by Zhao and Xie [24]. The data in [24] and the respective illustration [25, p. 5] show at least four sharper peaks, where each peak was achieved during the great unifying dynasties. Indeed, our DWM can produce oscillating/periodic population numbers with sharper peaks in the population numbers; see Fig 11. Furthermore, our DWM can also produce transient oscillations of varying amplitude in quite a parameter regions as one may approach a limit cycle or approach a weakly-stable spiral equilibrium; see also Figs 8–10. Yet, in summary it is very important to point again that an *exact* matching to a particular historical situation would require a reduction of the number of parameters via a detailed statistical fitting. Indeed, if we have too many free parameters, then the number of dynamical possibilities is too large and one encounters a classical ‘overfitting’ phenomenon. Unfortunately, it is in general very difficult to have a good estimator for all relevant parameters, even for relatively simple models, from historical data. Hence, one is currently limited to qualitative explanations and the discovery of general effects.

## 4 Conclusion

One should always have in mind that the “Demographic-Wealth model” (DWM), as well as the most other models, provides a simplified view of state and population dynamics and only takes into account few fundamental drivers; in particular, there are certainly more complex models that have been developed [26–28]. However, the DWM can be seen as an useful alternative or variant of the “Demographic-Fiscal model” (DFM). The advantage of the DWM is that the fiscal component is not the only and most important one of the model. Other parameters like the carrying capacity, the state expenditures or the wealth gain play a significant role too. Furthermore, the DWM still remains tractable in terms of direct mathematical analysis, which is often not the case for models of high(er) complexity.

In addition, the model is supported by the theories of Khaldun, Goldstone and Olson. These theories can also explain partly the appearance of Hopf bifurcations and the according limit cycle behavior. In particular, we have seen that the DWM can show very reasonable and quite realistic behaviour such as coexistence, bifurcations and periodicity. These can be interpreted within the framework of the theories we outlined initially. Furthermore, we can link the DWM to the paradox of enrichment as well to secular cycles. Yet, the DWM is still relatively simple algebraically allowing for analytical results as well as fast numerical continuation runs.

Of course, many further extensions of the DWM could be developed. For example, adding class structure would be a meaningful development of the model, although it would make the model more complicated. Turchin himself, as well as Goldstone, state that class structure, respectively elites, play an important role in state dynamics, that is, why Turchin extended the DFM with a class structure to have a more realistic view on state dynamics. For the DWM it would be interesting to see how class structure would affect the behavior of the system. Especially the effect on the appearance of Hopf bifurcations could be of special interest. In addition, studying different nonlinearities might be a very useful next step. For example, this may include additional nonlinear saturation effects for taxation, which we have not considered as already the population saturation is enough together with the coupling structure to produce interesting and practically relevant bounded orbits such as secular cycles.

## Appendix A. Proof of Theorem 4.1

*Proof*. We follow the ideas provided in [17, p. 111ff]. Let *g*(*N*, *S*), *p*(*N*) and *q*(*N*) be as defined in Subsection 3.2. The following constraints hold:

- There exists a *k*_0_ > 0, such that *g*(*N*, *S*) > 0 if 0 < *N* < *k*_0_ + *cS* and *S* ≥ 0, *g*(*k*_0_, 0) = 0, and *g*(*N*, *S*) < 0 if *N* > *k*_0_+ *cS*.
- *p*(0) = 0 and *p*(*N*) > 0 for positive *N*.
- *q*(0) = 0, *q*(*N*) > 0, *q*′(*N*) > 0 if *N* > 0.

The nullclines for *S* are

$$\begin{array}{c} S=0,\;l_{1}=\left\{\left(N,S\right):N=\hat{N},q\left(\hat{N}\right)=d\right\} \end{array}$$

and due to the conditions on *q* there is a unique point $\hat{N}$. For *N* it holds for the nullclines that

$$\begin{array}{c} N=0,\;l_{2}=\{\left(N,S\right):S=\frac{Ng(N,S)}{p(N)}\}. \end{array}$$

Due to the fact that the axes are composed of the orbits, $R_{+}^{2}$ is invariant. Two cases are possible. In the first case, assume that N* < *k*_0_ + cS*, then in $R_{+}^{2}$ there are three equilibria

$$\begin{array}{c} {\hat{x}}_{0}=\left(0,0\right),\;{\hat{x}}_{1}=\left(k_{0},0\right),\;{\hat{x}}_{2}=\left(N^{*},S^{*}\right), \end{array}$$

where

$$\begin{array}{c} S^{*}=\frac{N^{*}g\left(N^{*},S^{*}\right)}{p(N^{*})}. \end{array}$$

In the case N* > *k*_0_ + cS* only ${\hat{x}}_{0}$ and ${\hat{x}}_{1}$ are part of $R_{+}^{2}$. For the Jacobian one gets

$$\begin{array}{c} J\left(N,S\right)=[\begin{array}{cc} g\left(N,S\right)+N\frac{\partial }{\partial N}g\left(N,S\right)-Sp^{\prime }\left(N\right) & N\frac{\partial }{\partial S}g\left(N,S\right)-p\left(N\right)\\ Sq^{\prime }\left(N\right) & -d+q(N) \end{array}]. \end{array}$$

For ${\hat{x}}_{0}$ this yields

$$\begin{array}{c} J\left({\hat{x}}_{0}\right)=[\begin{array}{cc} g(0,0) & 0\\ 0 & -d \end{array}]. \end{array}$$

Therefore, ${\hat{x}}_{0}$ is always a saddle point, with the stable manifold on *S*-axis and unstable manifold on *N*-axis.

The equilibrium ${\hat{x}}_{1}$ can either be stable or unstable. Indeed, the Jacobian yields

$$\begin{array}{c} J\left({\hat{x}}_{1}\right)=[\begin{array}{cc} k_{0}g^{\prime }\left(k_{0},0\right) & k_{0}\frac{\partial }{\partial S}g\left(k_{0},0\right)-p\left(k_{0}\right)\\ 0 & -d+q(k_{0}) \end{array}]. \end{array}$$

Due to the assumptions, *g*′(*k*_0_, 0) < 0, therefore, if *q*(*k*_0_) > *d* then (*k*_0_, 0) is a saddle point, and if *q*(*k*_0_) < *d* then (*k*_0_, 0) is a stable node. If (*k*_0_, 0) is a saddle, then, according to the analysis, also $\left(N^{*},S^{*}\right)\in R_{+}^{2}$. Here, the Jacobian yields

$$\begin{array}{cc} J({\hat{x}}_{2}) & =[\begin{array}{cc} g\left(N^{*},S^{*}\right)+N^{*}\frac{\partial }{\partial N}g\left(N^{*},S^{*}\right)-S^{*}p^{\prime }\left(N^{*}\right) & N^{*}\frac{\partial }{\partial S}g\left(N^{*},S^{*}\right)-p\left(N^{*}\right)\\ S^{*}q^{\prime }\left(N^{*}\right) & 0 \end{array}]\\ & =[\begin{array}{cc} \mu (N^{*},S^{*}) & N^{*}\frac{\partial }{\partial S}g\left(N^{*},S^{*}\right)-p\left(N^{*}\right)\\ S^{*}q^{\prime }\left(N^{*}\right) & 0 \end{array}] \end{array}$$

where

$$\begin{array}{c} \mu \left(N^{*},S^{*}\right)=p\left(N\right)\frac{\partial }{\partial N}(\frac{Ng(N,S^{*})}{p(N)}){|}_{N=N^{*}}. \end{array}$$

Therefore, it holds that

$$\begin{array}{c} \text{tr}\left(J\left({\hat{x}}_{2}\right)\right)=\mu \left(N^{*},S^{*}\right),\;\text{det}\left(J\left({\hat{x}}_{2}\right)\right)=-S^{*}q^{\prime }\left(N^{*}\right)(N^{*}\frac{\partial }{\partial S}g\left(N^{*},S^{*}\right)-p\left(N^{*}\right)). \end{array}$$

For a possible stable equilibrium ${\hat{x}}_{2}$ it is required that

$$\begin{array}{c} p\left(N^{*}\right)>N^{*}\frac{\partial }{\partial S}g\left(N^{*},S^{*}\right). \end{array}$$

If this condition holds, the sign of $\text{tr}(J\left({\hat{x}}_{2}\right))$ is determined by the slope of the tangent line to *l*_2_ at N*. If this slope is positive, then ${\hat{x}}_{2}$ is an unstable node or focus. This implies that there exists a small neighborhood $N({\hat{x}}_{2})$ of ${\hat{x}}_{2}$ which is negative invariant (the orbits leave this neighborhood with *t* > 0). Next, consider the unstable manifold of ${\hat{x}}_{1}$. It emanates from ${\hat{x}}_{1}$ in the region where $\dot{N}<0,\;\dot{S}>0$ and therefore it has to reach the nullcline *N* = N*, named *B*_1_. After *B*_1_ $\dot{N}<0$ and $\dot{S}<0$, therefore the orbit has to cross *l*_2_ at the point *B*_2_.

Now the domain is $\dot{N}>0,\dot{S}<0$, which means that the orbit has to cross *l*_1_ again at the point *B*_3_. Let *B*_4_ = (N*, 0). Consider the closed path ${\hat{x}}_{1},\;B_{1},\;B_{2},\;B_{3},\;B_{1}$, and ${\hat{x}}_{1}$. Define *G* as the domain confined by this path without $N({\hat{x}}_{2})$.

*G* is positive invariant as the set whose boundaries are either orbits or the line on which the direction of the phase flow points “inwards” of *G*. By construction, *G* does not possess equilibria and hence, by the Poincaré-Bendixson theory, see [29] for details, it must contain at least one closed curve, which has to be an *ω*-limit set for the orbits entering *G*. Assume that N*, S* satisfy *μ*(N*, S*) < 0. Then ${\hat{x}}_{2}$ is stable. If N* is decreased to the point where the slope of *l*_2_ is zero, then *μ*(N*, S*) = 0, and there is a non-hyperbolic equilibrium ${\hat{x}}_{2}$ with two purely imaginary eigenvalues. As for the DWM *μ* is also dependent on *S*, a similar situation as for N* can happen with S*. This means that there could be a possibility where S* is changed to a point where also *μ*(N*, S*) = 0. This point corresponds to a Hopf bifurcation of ${\hat{x}}_{2}$.

## Appendix B. Proof of Theorem 4.2

*Proof*. We use the same steps as in the proof provided in Appendix A with the difference that *c* = 0 and therefore the function *g*(*N*, *S*) only depends on *N*. For the equilibria (0, 0) and (*k*_0_, 0) the conditions remain the same, (0, 0) is always a saddle and (*k*_0_, 0) is a stable node if *q*(*k*_0_) < *d* and a saddle if *q*(*k*_0_) > *d*. In the case of a saddle, also $\left(N^{*},S^{*}\right)\in R_{+}^{2}$. Here, the Jacobian yields

$$\begin{array}{cc} J({\hat{x}}_{2}) & =[\begin{array}{cc} g\left(N^{*}\right)+N^{*}g^{\prime }\left(N^{*}\right)-S^{*}p^{\prime }\left(N^{*}\right) & -p(N^{*})\\ S^{*}q^{\prime }\left(N^{*}\right) & 0 \end{array}]=[\begin{array}{cc} \mu (N^{*}) & -p(N^{*})\\ S^{*}q^{\prime }\left(N^{*}\right) & 0 \end{array}] \end{array}$$

where

$$\begin{array}{c} \mu \left(N^{*}\right)=p\left(N\right)(\frac{Ng(N)}{p(N)}{)}^{\prime }{|}_{N=N^{*}}. \end{array}$$

Therefore, it holds that

$$\begin{array}{c} \text{tr}\left(J\left({\hat{x}}_{2}\right)\right)=\mu \left(N^{*}\right),\;\text{det}\left(J\left({\hat{x}}_{2}\right)\right)=S^{*}q^{\prime }\left(N^{*}\right)p\left(N^{*}\right)>0. \end{array}$$

Following again the argumentation of the proof above and assuming that N* satisfies *μ*(N*) < 0, Then ${\hat{x}}_{2}$ is stable. If N* is decreased to the point where the slope of *l*_2_ is zero, then *μ*(N*) = 0, and there is a non-hyperbolic equilibrium ${\hat{x}}_{2}$ with two purely imaginary eigenvalues. This point corresponds to a Hopf bifurcation of ${\hat{x}}_{2}$.

For the stability of the coexistence point it is necessary that $\text{tr}(J\left({\hat{x}}_{2}\right))<0$, otherwise a Hopf bifurcation occurs if the trace equals zero (limit cycle if greater than zero). As the limit cycle behavior is more interesting, the following has to be satisfied,

$$\begin{array}{cc} \text{tr}(J\left({\hat{x}}_{2}\right)) & >0\\ \Leftrightarrow \;\;0 & <(rN^{*}(\frac{1}{d+N^{*}}-\frac{N^{*}}{k_{0}\left(d+N^{*}\right)}-\frac{1}{k_{0}})\\ \Leftrightarrow \;\;\frac{1}{d+N^{*}} & >(\frac{N^{*}}{k_{0}\left(d+N^{*}\right)}+\frac{1}{k_{0}})\\ \Leftrightarrow \;\;\frac{1}{d+N^{*}} & >\frac{2N^{*}+d}{k_{0}\left(d+N^{*}\right)}\\ \Leftrightarrow \;\;k_{0} & >2\frac{\beta }{g}+d. \end{array}$$

This yields the equations for the different parameters,

$$\begin{array}{c} \begin{array}{c} k_{0}>2\frac{\beta }{g}+d,\;\;\;\;d<k_{0}-2\frac{\beta }{g},\;\;\;\;\beta <\frac{1}{2}\left(k_{0}-d\right)g,\;\;\;\;g>\frac{2\beta }{k_{0}-d}. \end{array} \end{array}$$

The assumptions at the beginning of Theorem 4.2 are needed for the existence of the Hopf bifurcations. For $\frac{\beta }{g}<k_{0}\leq 2\frac{\beta }{g}$ there cannot be a Hopf bifurcation for the parameter *d*, because the equation cannot be fulfilled for *d* > 0. For *d* ≥ *k*_0_, there cannot be a Hopf bifurcation for the parameters *g* and *β*, because the equations cannot be fulfilled for *β* > 0, *g* > 0.
